# Prediction and risk stratification from hospital discharge records based on Hierarchical sLDA

**DOI:** 10.1186/s12911-022-01747-3

**Published:** 2022-01-15

**Authors:** Guanglei Yu, Linlin Zhang, Ying Zhang, Jiaqi Zhou, Tao Zhang, Xuehua Bi

**Affiliations:** 1 School of Medical Engineering and Technology, Xinjiang Medical University, No.567 North Shangde Road, Urumqi, China; 2 College of Information Science and Engineering, Xinjiang University, Urumqi, China; 3 The First Affiliated Hospital of Xinjiang Medical University, Urumqi, China

**Keywords:** Risk stratification, Topic models, Supervised latent Dirichlet allocation, Hospital discharge records

## Abstract

**Background:**

The greatly accelerated development of information technology has conveniently provided adoption for risk stratification, which means more beneficial for both patients and clinicians. Risk stratification offers accurate individualized prevention and therapeutic decision making etc. Hospital discharge records (HDRs) routinely include accurate conclusions of diagnoses of the patients. For this reason, in this paper, we propose an improved model for risk stratification in a supervised fashion by exploring HDRs about coronary heart disease (CHD).

**Methods:**

We introduced an improved four-layer supervised latent Dirichlet allocation (sLDA) approach called Hierarchical sLDA model, which categorized patient features in HDRs as patient feature-value pairs in one-hot way according to clinical guidelines for lab test of CHD. To address the data missing and imbalance problem, RFs and SMOTE methods are used respectively. After TF-IDF processing of datasets, variational Bayes expectation-maximization method and generalized linear model were used to recognize the latent clinical state of a patient, i.e., risk stratification, as well as to predict CHD. Accuracy, macro-F1, training and testing time performance were used to evaluate the performance of our model.

**Results:**

According to the characteristics of our datasets, i.e., patient feature-value pairs, we construct a supervised topic model by adding one more Dirichlet distribution hyperparameter to sLDA. Compared with established supervised algorithm Multi-class sLDA model, we demonstrate that our proposed approach enhances training time by 59.74% and testing time by 25.58% but almost no loss of average prediction accuracy on our datasets.

**Conclusions:**

A model for risk stratification and prediction of CHD based on sLDA model was proposed. Experimental results show that Hierarchical sLDA model we proposed is competitive in time performance and accuracy. Hierarchical processing of patient features can significantly improve the disadvantages of low efficiency and time-consuming Gibbs sampling of sLDA model.

## Introduction

Cerebrovascular accident (CVA), coronary heart disease (CHD) and other cardiovascular diseases (CVD) are the leading causes of death and serious family burden in China nowadays. According to the World Health Organization (WHO), risk factors can increase the chances that a person suffers from that disease (WHO, 2014). Risk stratification incorporating these risk factors can be used by physicians to assess the risk of atherosclerotic of individual patient, such as taking treatment with drugs to lower blood pressure and blood cholesterol based on an individual’s absolute cardiovascular risk [1]. According to accurate risk stratification, so as to reduce the overall risk of CVD or CHD, physicians can formulate corresponding comprehensive clinical treatments or life intervention management programs for patients with different risk levels. To carry out risk stratification also plays an important role in individualized nursing, drug development and cost estimation of CVD [2].

In recent years, with the rapid development of medical information technology, the applications of electronic medical records (EMRs) are becoming more and more widely and in-depth. EMRs store and share clinical information of patients, such as general items, diagnostic images (e.g., X-rays), history of present illness (HPI), family history, lab results etc. for different diseases [3–5] and various medical applications [6–9], especially HDRs, which include abundant information on patient risk factors. In 2014, in order to identify and extract medical risk factors related to CHD, i2b2/UTHealth Natural Language Processing shared task with respect to the longitudinal medical records of patients with diabetes. The risk factors included hypertension, hyperlipidemia, obesity, smoking status, family history, diabetes [10].

Multivariable traditional assessment models have been provided to estimate CVD risk [11, 12]. At the cohort level, these risk stratification models take statistical analysis techniques (e.g., logistic regression, Cox regression, etc.) to estimate the absolute risk of patients, which offer little insight beyond a flat score-based segmentation that has high cost, carefully selected and highly stratified patient characteristics [13].

In this study, we proposed a four-layer probabilistic topic model, i.e., Hierarchical sLDA model, for risk stratifications and prediction of CHD by using the diagnosis cases from HDRs, where Hierarchical sLDA model is a variant of sLDA. Firstly, we collected data from real clinical settings and annotated risk factors with an annotation tool developed by ourselves, under the guidance of clinicians. Then, we extracted the patient feature-value pairs of risk factors and encoding them in One Hot way according to clinical guidelines for lab test of CHD. Meanwhile, to address the data missing and imbalance problem, RFs and SMOTE are used respectively. After TF-IDF processing of datasets, variational Bayes Expectation-maximization (VBEM) and generalized linear model (GLM) was used to recognize the latent clinical state of a patient, i.e., risk stratification, as well as to predict CHD. Experimental results show that our model can significantly improve the disadvantages of low efficiency and time-consuming of sLDA model.

## Related work

With the continuous digitization and storage of knowledge in the forms of news, blogs, web pages, scientific articles, books, images, sound, video and social networks, it is more and more difficult for us to find what we are looking for from massive information [14].

In 1999, Thomas Hofmann proposed probabilistic latent semantic indexing (PLSI), which characterizes the polysemy of a word by describing the word frequency vector with multinomial distribution [15]. The proposal of PLSI enables the discovery and analysis of potential topics or categories in a large number of documents, and realizes the tasks of document clustering and dimension reduction.

In order to overcome the defects of inconsistent generative semantics of PLSI that cannot generate (i.e., predict) new documents and its model overfitting, Blei et al. [16] proposed LDA (latent Dirichlet allocation) topic model in 2003, and two commonly used approximate inference methods are Variational Bayes (VB) deterministic and collapsed Gibbs sampling (GS) stochastic approximation. Girolami and Kabán [17] showed that PLSI is maximum a posteriori (MAP) estimated LDA model under uniform Dirichlet prior. LDA is a three-layer hierarchical (including documents, topics and words) Bayesian unsupervised topic model. Based on the Bag-of-words (BOW) representation, LDA can cluster topics or classify texts from a large number of documents and has good scalability. With the development of probabilistic topic model, it has made continuous progress in image analysis, bioinformatics and other fields [18]. Jelodar et al. [19] reviewed the research progress, future development trend and wide application subjects of LDA topic model from 2003 to 2016, such as Social Network, Crime Science, Medical/Biomedical and Linguistic science.

For text classification, Li and McCallum [20] showed that LDA model does not capture the correlation between topics, and the accuracy and efficiency of topic classification are not outstanding and insufficient. Furthermore, although LDA model can achieve document clustering and dimension reduction and other tasks, it is not suitable for prediction. In 2010, Blei and McAuliffe [21] proposed supervised latent Dirichlet allocation (sLDA). By combining GLM for latent topics, and selecting different exponential distribution family according to response variables, multiple response variables (e.g., real, category or multinomial response variable) can be predicted.

Wang et al. [22] extended to image classification and proposed Multi-class sLDA prediction model by selecting GLM model for multinomial response. Their experimental results showed that the average accuracy of the model for LabelMe datasets (1600 images, 8 categories) and UIUC-Sport datasets (1792 images, 8 categories) was 76% and 66% respectively, which was better than that of Li and Perona [23] and Bosch et al. [24], and the average accuracy increased by more than 10%.

By using factor graph to represent collapsed LDA to encode the joint probability, Zeng et al. [25] enabled the belief propagation (BP) for approximate inference and parameter estimation and has enhanced both speed and accuracy by experimental results on four document datasets.

Besides these methods, there are various variants and applications about the state-of-the-art topic model LDA recently, e.g., opinion mining on big data [26], stock market returns [27], topic change point detection [28], open-ended versus closed-ended response [29], etc.

Motivated by these observations, our study proposes a probabilistic ensemble classification method, which distinguishes from other methods in that: (1) it takes a slight structural change to standard sLDA, which improves three-layer sLDA to a four-layer called Hierarchically sLDA model, and achieves encouraging experimental results in terms of time performance; (2) our model can provide prediction of CHD and risk stratification simultaneously.

The remaining sections of this paper are organized as follows. “Hierarchical sLDA” section describes our proposed model, variational inference and parameter estimation. “Experiments” section carefully describes the datasets and presents our model experimental results. Finally, we present our conclusions possible directions for future work in “Conclusions” section.

## Hierarchical sLDA

### Modeling HDRs and labels

Firstly, we summarize some important symbols and notations in this paper shown in Table 1.

**Table 1:** Symbols and notations

Symbols	Notations
$1 \le d \le D$	HDRs index
$f_{1:F}$	Patient features
$v_{1:N}$	Patient feature-value pairs
$1 \le k \le K$	Topic index
$\pi _{k}$	Topic-feature multinomials of feature *f*, $\pi _{k}$ is *F*-dimensional vector
$\beta _{k}$	Topic-value multinomials of feature-value *v*, $\beta _{k}$ is *N*-dimensional vector
$\alpha$	*K*-dimensional Dirichlet parameter vector
$\theta$	*K*-dimensional topic proportions
$r_{1:N}$	Topic assignments
*y*	Response variables
$\eta _{1:C}$	Class coefficients

**Fig. 1:**
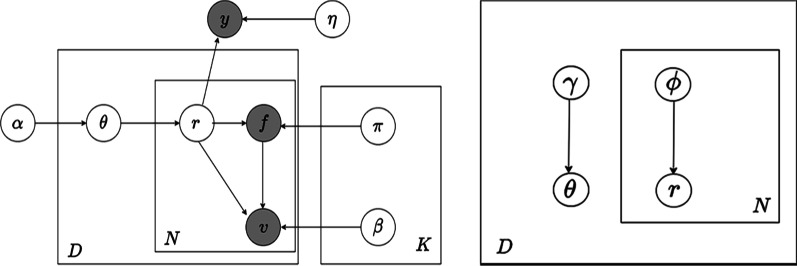
Probabilistic graphical model. The probabilistic graphical model representation of Hierarchical sLDA (left); the graphical model representation of variational distribution (right)

The graphical model representation of hierarchical sLDA is depicted in Fig. 1. Nodes are random variables; edges indicate possible dependence; a shaded node is an observed variable; an unshaded node is a hidden variable.

Each HDRs is represented as a bag of patient feature $f_{1:F}$ or patient feature-value pair $v_{1:N}$. The category *c* is a discrete class label. Each topic is a distribution over a vocabulary of patient feature, and also be regarded as distribution over vocabulary of patient feature-value pair. *K* is the number of latent topics; *N* is the number of feature-value of a single HDRs; *D* is the number of patient HDRs.

Our model assumes the following generative process of an HDRs, and its class label.

1. Draw topic proportions $\theta$, $\theta \mid \alpha \sim {\text {Dir}}(\alpha )$;
2. For each patient HDRs feature-value pair $v_{1:N}$:
  - Draw topic assignment $r_{n}$ from category distribution with parameter $\theta$, $r_{\mathrm {n}} \mid \theta \sim {\text {Mult}}(\theta )$;
  - Draw feature $f_{n}$ from category distribution with parameter $\pi$, $f_{n} \mid r_{n}, \pi _{1: K} \sim {\text {Mult}}\left( \pi _{r_{n}}\right)$;
  - Draw feature-value pair $v_{n}$ from category distribution with parameter $\beta$, $v_{n} \mid r_{n}, f_{n}, \beta _{1: K} \sim {\text {Mult}}\left( \beta _{f_{n}, r_{n}}\right)$;
3. Draw class label *y* from GLM distribution $y \mid r_{1: N}, \eta \sim GLM({\bar{r}}, \eta )$, where ${\bar{r}}=\frac{1}{N} \sum _{n=1}^{N} r_{n}$, that is:
  $$\begin{aligned}p\left( y \mid r_{1:N}, \eta \right) =\exp \left( \eta _{y}^{T} {\bar{r}}\right) / \sum _{l=1}^{C} \exp \left( \eta _{l}^{T} {\bar{r}}\right) \end{aligned}$$
  it also can be written as exponential distribution family:
  $$\begin{aligned} p\left( y \mid r_{\mathrm {1:N}}, \eta \right) =\exp \left\{ \eta _{y}^{T} {\bar{r}}-\log \left( \sum _{l=1}^{C} \exp \left( \eta _{l}^{T} {\bar{r}}\right) \right) \right\} \end{aligned}$$

The posterior inference of the model can be divided into three steps or tasks.

- *Variational approximate inference* A posterior inference is to compute the conditional distribution of the latent variables at the patient HDRs level. That is, approximate inference is used to estimate the parameters of the variational distribution.
- *Parameter estimation* The model parameters $\alpha , \beta _{1: K}, \pi _{1: K}, \eta$ are fitted with variational expectation maximization (EM) by maximum likelihood estimation.
- *Prediction and risk stratification* To perform prediction and risk stratification over the model parameters$\alpha , \beta _{1: K}, \pi _{1: K}, \eta$ and variational distribution parameters $\gamma , \phi$ means approximation of posterior expectation of response variable $y={\mathrm {E}}\left[ Y \mid r_{1:N}, \alpha , \beta _{1: K}, \pi _{1: K}, \eta \right]$.

### Variational approximate inference

Both Parameter estimation and prediction depend on the posterior inference. Following Jordan et al. [16], given patient HDRs and response variable *y*, we start the joint distribution in the following equation. The real posterior of latent variables (including topic proportions $\theta$, topic $r_{n}$) is:

$$\begin{aligned} \begin{aligned}{}&p\left( \theta , r_{1:N} \mid f_{1:N}, v_{1:N}, y, \alpha , \pi _{1: {\mathrm {K}}}, \beta _{1: K}, \eta \right) \\&\quad =\frac{p(\theta \mid \alpha )\left( \prod _{n=1}^{N} p\left( {\mathrm {r}}_{n} \mid \theta \right) p\left( f_{n} \mid {\mathrm {r}}_{n}, \pi _{1: {\mathrm {K}}}\right) p\left( v_{n} \mid {\mathrm {r}}_{n}, f_{n}, \beta _{1: K}\right) \right) p\left( y \mid r_{1:N}, \eta \right) }{\int p(\theta \mid \alpha ) d \theta \sum _{r_{1:{\mathrm {N}}}}\left( \prod _{n=1}^{N} p\left( {\mathrm {r}}_{n} \mid \theta \right) p\left( f_{n} \mid {\mathrm {r}}_{n}, \pi _{1: {\mathrm {K}}}\right) p\left( v_{n} \mid {\mathrm {r}}_{n}, f_{n}, \beta _{1: K}\right) \right) p\left( y \mid r_{1:N}, \eta \right) } \end{aligned} \end{aligned}$$

As the denominator of posterior distribution is difficult to calculate, we use mean-field variational approximation inference:

$$\begin{aligned} q\left( \theta , r_{1: N} \mid \gamma , \phi _{\mathrm {1: N}}\right) =q(\theta \mid \gamma ) \prod _{n=1}^{N} q\left( {\mathrm {r}}_{n} \mid \phi _{n}\right) \end{aligned}$$

Let $\zeta =\left\{ \alpha , \pi _{1: K}, \beta _{1: K}, \eta \right\}$, the *KL* divergence between the real posterior of latent variables $p\left( \theta , r_{1:N} \mid f_{1:N}, v_{1:N}, y, \zeta \right)$ and variational distribution $q\left( \theta , r_{\mathrm {1:N}}\right)$ is:

$$\begin{aligned} \begin{aligned}{}&\mathrm {D}\left( q\left( \theta , r_{1:N}\right) \Vert p\left( \theta , r_{1:N} \mid f_{1:N}, v_{1:N}, y, \zeta \right) \right) \\&\quad ={\mathrm {E}}_{q}\left[ \log q\left( \theta , r_{1:N}\right) \right] -{\mathrm {E}}_{q}\left[ \log p\left( \theta , r_{1:N} \mid f_{1:N}, v_{1:N}, y, \zeta \right) \right] \\&\quad ={\mathrm {E}}_{q}\left[ \log q\left( \theta , r_{1:N}\right) \right] -{\mathrm {E}}_{q}\left[ \log p\left( \theta , r_{1:N}, f_{1:N}, v_{1:N}, y \mid \zeta \right) \right] +\log p\left( f_{1:N}, v_{1:N}, y \mid \zeta \right) \\&\quad \ge 0 \end{aligned} \end{aligned}$$

So the evidence lower bound (*ELBO*) is:

$$\begin{aligned} \log p\left( f_{1: N}, v_{1: N}, y \mid \zeta \right) \ge {\mathrm {E}}_{q}\left[ \log p\left( \theta , r_{1: N}, f_{1: N}, v_{1: N}, y \mid \zeta \right) \right] -{\mathrm {E}}_{q}\left[ \log q\left( \theta , r_{1: N}\right) \right] \end{aligned}$$

we denote *ELBO* by ${\mathscr {L}}(\bullet )$, and the entropy of variational distribution by $H(q)=-{\mathrm {E}}_{q}\left[ \log q\left( \theta , r_{1: N}\right) \right]$, and then *ELBO* is written as:

1 $$\begin{aligned}{}&{\mathscr {L}}\left( f_{1: N}, v_{1: N}, y \mid \zeta \right) \nonumber \\&\quad ={\mathscr {L}}\left( \gamma , \phi _{1: N} \mid \zeta \right) ={\mathrm {E}}_{q}[\log p(\theta \mid \alpha )]+\sum _{n=1}^{N} {\mathrm {E}}_{q}\left[ \log p\left( \mathrm {r}_{n} \mid \theta \right) \right] \nonumber \\&\qquad +\,\sum _{n=1}^{N} {\mathrm {E}}_{q}\left[ \log p\left( f_{n} \mid \mathrm {r}_{n}, \pi _{1: \mathrm {K}}\right) \right] +\sum _{n=1}^{N} {\mathrm {E}}_{q}\left[ \log p\left( v_{n} \mid \mathrm {r}_{n}, f_{n}, \beta _{1: \mathrm {F}}\right) \right] \nonumber \\&\qquad +\,{\mathrm {E}}_{q}\left[ \log p\left( y \mid r_{1: N}, \eta \right) \right] +H(q) \end{aligned}$$

We fit these parameters by maximizing *ELBO* with respect to $\gamma ,\phi$ and obtain an estimate of the posterior under the sense of *KL* divergence between $q\left( \theta , r_{1: N}\right)$ and the true posterior $p\left( \theta , r_{1: N} \mid f_{1: N}, v_{1: N}, y, \zeta \right)$.

The terms of equation 1 are as follows:

2 $$\begin{aligned} {\mathrm {E}}_{q}[\log p(\theta \mid \alpha )]= & {} \log \Gamma \left( \sum _{i=1}^{K} \alpha _{i}\right) -\sum _{i=1}^{K} \log \Gamma \left( \alpha _{i}\right) \nonumber \\&+\sum _{i=1}^{K}\left( \alpha _{i}-1\right) E_{q}\left[ \log \theta _{i}\right] \end{aligned}$$

3 $$\begin{aligned} \sum _{n=1}^{N} {\mathrm {E}}_{q}\left[ \log p\left( \mathrm {r}_{n} \mid \theta \right) \right]= & {} \sum _{\mathrm {n}=1}^{N} \sum _{i=1}^{K} \varphi _{n, i} {\mathrm {E}}_{q\left( \theta _{i} \mid \gamma \right) }\left[ \log \theta _{i}\right] \end{aligned}$$

4 $$\begin{aligned} \sum _{n=1}^{N} {\mathrm {E}}_{q}\left[ \log p\left( f_{n} \mid \mathrm {r}_{n}, \pi _{1: \mathrm {K}}\right) \right]= & {} \sum _{n=1}^{N} \sum _{i=1}^{K} \phi _{n, i} \log \pi _{i, f_{n}} \end{aligned}$$

5 $$\begin{aligned} \sum _{n=1}^{N} {\mathrm {E}}_{q}\left[ \log p\left( v_{n} \mid \mathrm {r}_{n}, f_{n}, \beta _{1: \mathrm {k}}\right) \right]= & {} \sum _{n=1}^{N} \sum _{i=1}^{K} \phi _{n, i} \log \beta _{i, v_{n}} \end{aligned}$$

6 $$\begin{aligned} E_{q}\left[ \log p\right. \left. \left( y \mid r_{1: N}, \eta \right) \right]= & \,\eta _{y}^{T} \frac{1}{N} \sum _{n=1}^{N} \phi _{n}-E_{q}\left[ \log \sum _{l=1}^{C} \exp \left( \eta _{l}^{T} \overline{\mathrm {r}}\right) \right] \end{aligned}$$

7 $$\begin{aligned} H(q)= & {} -\log \Gamma \left( \sum _{i=1}^{K} \gamma _{i}\right) +\sum _{i=1}^{K} \log \Gamma \left( \gamma _{i}\right) \nonumber \\&-\sum _{i=1}^{K}\left( \gamma _{i}-1\right) \left( \Psi \left( \gamma _{i}\right) -\Psi \left( \sum _{l=1}^{K} \gamma _{l}\right) \right) \nonumber \\&-\sum _{n=1}^{N} \sum _{i=1}^{K} \phi _{n, i} \log \phi _{n, i} \end{aligned}$$

where $\Psi (\bullet )$ denotes the digamma function.

**Variational E-step**

The coordinate ascent method updates for variational parameter $\gamma$, which is the same as sLDA, does not directly involve the response variable *y*.

8 $$\begin{aligned} \gamma ^{new} \leftarrow \alpha +\sum _{n=1}^{N} \phi _{n} \end{aligned}$$

Under the conditions $E[\overline{\mathrm {r}}]={\bar{\phi }}=\frac{1}{N} \sum _{n=1}^{N} \phi _{n}$, the terms in *ELBO* containing $\phi _{n}$ are:

$$\begin{aligned} {\mathscr {L}}_{\left[ \phi _{n}\right] }&=\sum _{i=1}^{K} \phi _{n, i} E_{q}\left[ \log \theta _{i}\right] +\sum _{i=1}^{K} \phi _{n, i} \log \pi _{i, f_{n}}\\&\quad +\sum _{i=1}^{K} \phi _{n, i} \log \beta _{i, v_{n}}-\sum _{i=1}^{K} \phi _{n, i} \log \phi _{n, i}\\&\quad +\frac{1}{N} \sum _{i=1}^{K} \eta _{y, i} \phi _{n, i}-\log \left( \sum _{l=1}^{C} \prod _{n=1}^{N}\left( \sum _{i=1}^{K} \phi _{n, i} \exp \left( \eta _{l, i} \frac{1}{N}\right) \right) \right) \end{aligned}$$

Following Wang et al. [22], under the constraint $\sum _{i=1}^{K} \phi _{n, i}=1$ and setting partial derivatives to zero of the *ELBO* with respect to $\phi _{n, i}$, we write $\log \left( \sum _{l=1}^{C} \prod _{n=1}^{N}\right.$ $\left. \left( \sum _{i=1}^{K} \phi _{n, i} \exp \left( \eta _{l, i} \frac{1}{N}\right) \right) \right)$ as $h^{T} \phi _{n}$, and then obtain:

9 $$\begin{aligned} \phi _{n, i} \propto \pi _{i, f_{n}} \beta _{i, v_{n}} \exp \left( \Psi ^{\prime }\left( \gamma _{i}\right) +\frac{\eta _{y, i}}{N}-\left( h^{T} \phi _{n}^{o l d}\right) ^{-1} h_{i}\right) \end{aligned}$$

The variational EM algorithm alternatively updates Eqs. 8 and 9 until the bound on the expected log likelihood converges.

### Parameter estimation

Variational M-step is an optimization of *ELBO* on the whole HDRs datasets level w.r.t model parameters $\zeta =\left\{ \alpha , \pi _{1: K}, \beta _{1: K}, \eta \right\}$. Repeating the Variational E-step *D* times, we can obtain approximate posterior over latent variables $\theta , r_{1:N}$ for each patient HDRs. It is noted that different patient HDRs has different variational distributions $q_{d}\left( \theta , r_{1: N}\right)$. Then we obtain:

$$\begin{aligned} L\left( \alpha , \pi _{1: \mathrm {K}}, \beta _{1: K}, \eta ; D\right) =\sum _{\mathrm {d}=1}^{D}\left\{ E_{q_{d}}\left[ \log p\left( \theta _{d}, \mathrm {r}_{d, 1: N}, f_{d, 1: N}, v_{d, 1: N}, y_{d}\right) \right] +H\left( q_{d}\right) \right\} \end{aligned}$$

**Variational M-step**

Similarly, the coordinate ascent method is used to maximize the whole HDRs datasets *ELBO* to estimate the model parameters $\zeta =\left\{ \alpha , \pi _{1: K}, \beta _{1: K}, \eta \right\}$.

- Setting
  $$\begin{aligned}&\partial L\left( \alpha , \pi _{1: \mathrm {K}}, \beta _{1: \mathrm {K}}, \eta ; D\right) / \partial \pi _{k, f}=0\\&\partial L\left( \alpha , \pi _{1: \mathrm {K}}, \beta _{1: \mathrm {K}}, \eta ; D\right) / \partial \beta _{k, \mathrm {v}}=0 \end{aligned}$$
  it leads to:
  10 $$\begin{aligned} {\hat{\pi }}_{k, f}^{new}&\propto \sum _{d=1}^{D} \sum _{n=1}^{N} I\left( f=f_{n, k}^{d}\right) \phi _{n, k}^{d} \end{aligned}$$
  11 $$\begin{aligned} {\hat{\beta }}_{k, v}^{new}&\propto \sum _{d=1}^{D} \sum _{n=1}^{N} I\left( v=v_{n, k}^{d}\right) \phi _{n, k}^{d} \end{aligned}$$
- The whole HDRs datasets *ELBO* containing $\eta _{c}$ are:
  $$\begin{aligned} L_{\left[ \eta _{1:C}\right] }(\mathrm {D})=\sum _{d=1}^{D}\left( \eta _{c_{d}}^{T} {\bar{\phi }}_{d}-\log \left( \sum _{l=1}^{C}\left\{ \prod _{n=1}^{N}\left( \sum _{i=1}^{K} \phi _{n, i}^{d} \exp \left( \eta _{l, i} \frac{1}{N}\right) \right) \right\} \right) \right) \end{aligned}$$
  Setting $\frac{\partial L_{\left[ \eta _{1:C}\right] }(\mathrm {D})}{\partial \eta _{\mathrm {c}, i}}=0$ does not lead to a closed-form solution. Following Wang et al. [22], we optimize with conjugate gradient. Let $\kappa _{d}=\sum _{l=1}^{C}\left\{ \prod _{n=1}^{N} \phi _{n, \mathrm {r}_{n}} \right.$ $\left. \sum _{\mathrm {i}=1}^{K}\left( \exp \left( \eta _{l, i} \frac{1}{N}\right) \right) \right\}$, the derivatives are:
  $$\begin{aligned} \begin{aligned} \frac{\partial L_{\left[ \eta _{\mathrm {1:c}}\right] }(\mathrm {D})}{\partial \eta _{\mathrm {c}, i}}&=\sum _{d=1}^{D}\left( 1\left[ \mathrm {c}_{d}=\mathrm {c}\right] {\bar{\phi }}_{d, i}\right) -\sum _{d=1}^{D}\left( \kappa _{d}^{-1} \prod _{n=1}^{N}\left( \sum _{j=1}^{K} \phi _{n, \mathrm {j}}^{\mathrm {d}} \exp \left( \eta _{\mathrm {c}_{j}} \frac{1}{N}\right) \right) \right. \\&\quad \left. \times \sum _{n=1}^{N}\left( \frac{\frac{1}{N} \phi _{n, \mathrm {i}}^{\mathrm {d}} \exp \left( \eta _{c_{i}} \frac{1}{N}\right) }{\sum _{j=1}^{K} \phi _{n, \mathrm {j}}^{\mathrm {d}} \exp \left( \eta _{\mathrm {c}_{j}} \frac{1}{N}\right) }\right) \right) \end{aligned} \end{aligned}$$
- In this paper, We will address this setting in details about the Dirichlet parameter $\alpha$ and $\beta$ in “Discussion” section.

### Prediction and risk stratification

Under the fitted model$\left\{ \alpha ,\gamma ,\phi _{1:N},\pi _{1: K}, \beta _{1: K}, \eta \right\}$, the expected response value is:

$$\begin{aligned} E\left[ Y \mid f_{1: N},v_{1: N}, \alpha , \pi _{1: K}, \beta _{1: K}, \eta \right] =E\left[ \mu \left( \eta ^{\top } {\bar{r}}\right) \mid f_{1: N},v_{1: N}, \alpha , \pi _{1: K}, \beta _{1: K}\right] \end{aligned}$$

where $$\mu (\bullet )={\mathrm {E}}_{G L M}[Y \mid \cdot ]=\left[ \frac{\exp \left( \eta _{1}^{T} \overline{\mathrm {r}}\right) }{\sum _{l=1}^{C} \exp \left( \eta _{l}^{T} \overline{\mathrm {r}}\right) }, \ldots , \frac{\exp \left( \eta _{C}^{T} \overline{\mathrm {r}}\right) }{\sum _{l=1}^{C} \exp \left( \eta _{l}^{T} \mathrm {r}\right) }\right] ^{T}$$.

In classification, to estimate the probability of the label c with the variational distribution, we obtain:

$$\begin{aligned} \begin{aligned} E\left[ Y \mid v_{1: N}, \alpha , \pi _{1:K}, \right. \left. \beta _{1: K}, \eta \right]&\approx \int \exp \left( \log \frac{\exp \left( \eta _{c}^{T} \overline{\mathrm {r}}\right) }{\sum _{l=1}^{C} \exp \left( \eta _{l}^{T} \mathrm {r}\right) } q(r) \right) \mathrm {dr} \\&\ge \exp \left( {\mathrm {E}}_{q}\left[ \eta _{c}^{T} \overline{r}\right] -{\mathrm {E}}_{q}\left[ \log \left( \sum _{l=1}^{C} \exp \left( \eta _{l}^{T} {{\overline{r}}}\right) \right) \right] \right) \end{aligned} \end{aligned}$$

Thus, the prediction formulation is:

12 $$\begin{aligned} \mathrm {c}^{*}=\arg \max _{c \in \{1, \ldots , \mathrm {C}\}} {\mathrm {E}}_{q}\left[ \eta _{c}^{T} {{\overline{r}}}\right] =\arg \max _{c \in \{1, \ldots , \mathrm {C}\}} \eta _{c}^{T} {\bar{\phi }} \end{aligned}$$

where $$E[{{\overline{r}}}]={\bar{\phi }}=\frac{1}{N} \sum _{n=1}^{N} \phi _{n}$$.

## Experiments

### Methods

#### Data source

**Fig. 2:**
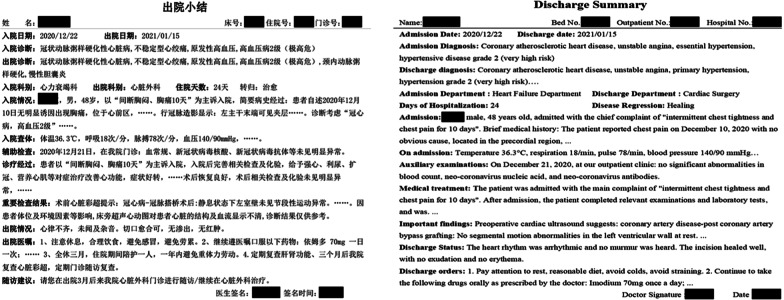
Original HDR. The original HDR in Chinese (left); The corresponding English version (right)

We propose our model on real-world datasets in the clinical domains collected from the Cardiology Department of the First Affiliated Hospital of Xinjiang Medical University containing 420 HDRs of CHD patients. These HDRs describe basic information about patients with a first diagnosis of coronary atherosclerotic heart disease, such as admission, treatment history, important test results, discharge status, discharge orders, and follow-up recommendations. An original HDR of a patient was described in Fig. 2-left, corresponding English version was shown in Fig. 2-right. In this paper, the patient’s diagnostic results is used as the class label for the data, including the following 4 classes: Stable Angina Pectoris(SAP), Unstable Angina Pectoris(UAP), Ischemic Cardiomyopathy(ICM) and Acute Miocardial Infarction(AMI).

#### Annotation of features

HDRs are a kind of unstructured text. In order to guarantee the accuracy of datasets and ensure the credibility as far as possible, each patient case in the experimental datasets was identified and evaluated by clinicians. This is a labor-intensive and laborious task.

**Fig. 3:**
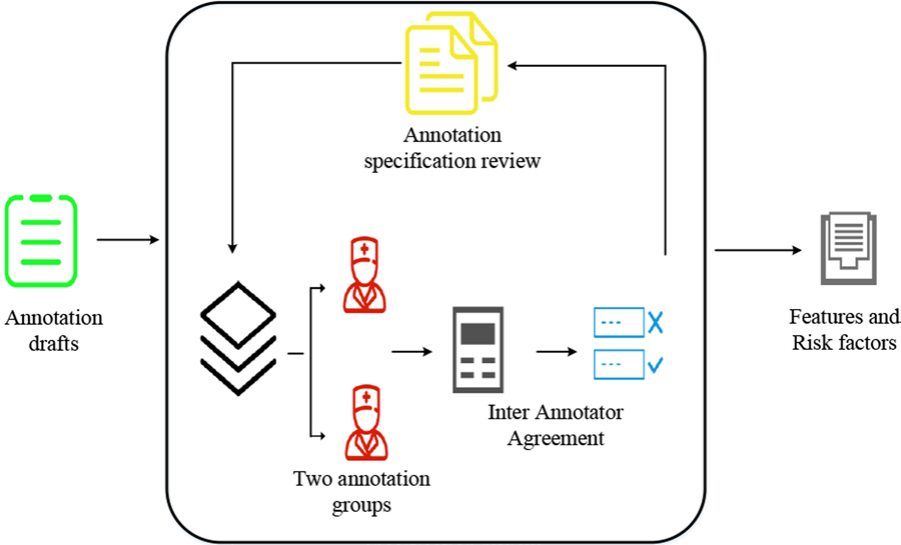
Features annotation of HDRs. The process of features annotation of HDRs

With an annotation tool developed by ourselves, we finished the features annotation of patients and obtain structured data including patient demographics, general items, history of present illness (HPI), laboratory results, conclusions of diagnoses etc., which provide a comprehensive source for risk stratification. Based on the CHD risk factors formulated in I2B2 and combined with the clinical experience of clinicians in the diagnosis and treatment, we drew up annotation criterion and guidelines for this study under the help of clinicians. This work consists of three pre-annotation and a formal annotation, with 50 HDRs randomly selected each time. The result was verified by Inter Annotator Agreement (IAA) after each annotation to guarantee the qualification. At the same time, the criterion and guidelines of annotation were constantly updated to ensure the standardization throughout the process. Figure 3 shows the features annotation process of HDRs.

#### Data preprocessing

While random forests (RFs) [30], which allow for avoiding over-fitting makes it suitable for processing data with outliers, missing values, have been widely applied to other fields such as biological prediction [31], we use RFs for filling the missing data.

And then, we obtain 34 patient features and 79 patient feature-value pairs, according to specific medical guidelines and specifications of CHD, by dividing patient features into a series of categories. Using the TF-IDF text mining technique to assign a weight to each feature and feature-value pair term, features matrix of 420 × 34 dimensions and feature-value pairs matrix of 420 × 79 dimensions are obtained respectively as the source datasets for modeling.

At the same time, 265 out of the 420 patient HDRs cases in the datasets are Stable Angina Pectoris (63.10%), and the class imbalance problem is encountered. In this paper, we use the SMOTE algorithm to generate additional samples from randomly oversampling minority class, and finally totaling 1060 samples are obtained. Therefore, features and diagnosis results can be extracted from HDRs, which provide data preparation for supervised training and testing and can be used as the labels of CHD. Summary statistics of the datasets are shown in Table 2.

**Table 2:** Summary statistics of datasets

Number of patient records		Number of patient features		Number of patient feature-value pairs
420		34		79

Top 15 risk factors		Frequency	Ratio (%)	Descriptions
Heart rhythm-Sinus		404	96.19%	
Antiplatelet medication-Yes		393	93.57	
Heart rate-norm.		371	88.33	
Lipid-lowering medication-Yes		369	87.86	
Chest pain-1$^{\star }$		338	80.48	1. Oppressive, stuffy or $\hbox {constrictive}^{\star }$ 2. Dyspnea
Range of Chest pain-2$^{\star }$		306	72.86	1. Located behind the sternal body 2. Affected precordial area, palm size $\hbox {range}^{\star }$ 3. Radiation to the left shoulder, left arm medial ring finger and little finger
Sex-Male		288	68.57	
Cardiac B-Ultrasound-Abn.		279	66.43	
Hypertension-Yes		271	64.52	
Ethnic-Han		253	60.24	
Incentive-Yes		224	53.33	
LDL-C-Abn.		253	60.24	
PCI or CABG-Yes		223	53.10	PCI: percutaneous coronary intervention CABG: coronary artery bypass grafting
$\beta$ blocker medications-Yes		170	40.48	
Carotid atherosclerosis with plaque-2$^{\star }$		164	39.05	1. No atherosclerotic plaque 2. Single plaque $\hbox {group}^{\star }$ 3. Multiple plaque group
Types of CHD	SAP	265	63.10	
	UAP	98	23.33	
	ICM	12	2.86	
	AMI	45	10.71	

### Results

For risk stratification and classification prediction of CHD, we use fivefold cross-validation method to create the train and test sets. Results are reported as an average across folds.

#### Classification performance

In order to perform assessment of the classification performance of our model, we compared it with previous salient approach Multi-class sLDA [22]. Multi-class sLDA embeds single softmax into LDA model, and reports better classification performance. The distinguishing factor between Hierarchical sLDA and Multi-class sLDA is the additional structure imposed on the feature-value pair, which would result in an outstanding performance in predictive performance.

**Fig. 4:**
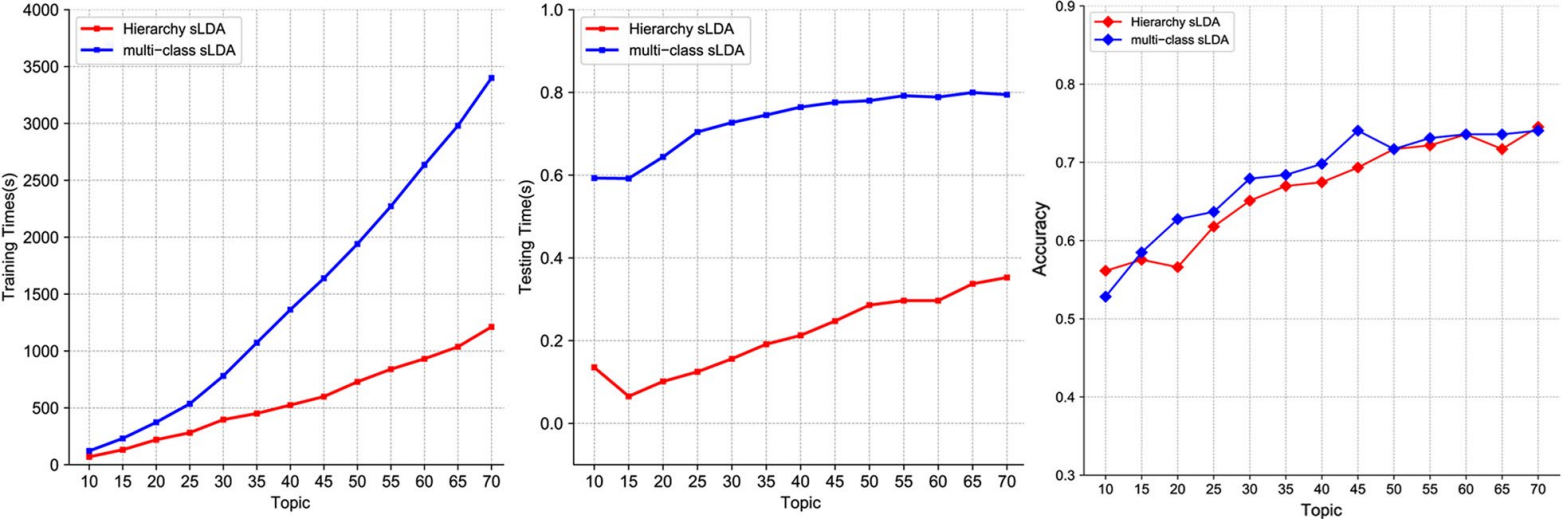
Comparison of performance. Comparison of over all classes based on fivefold cross validation: training time (left); testing time (middle); average accuracy (right)

**Fig. 5:**
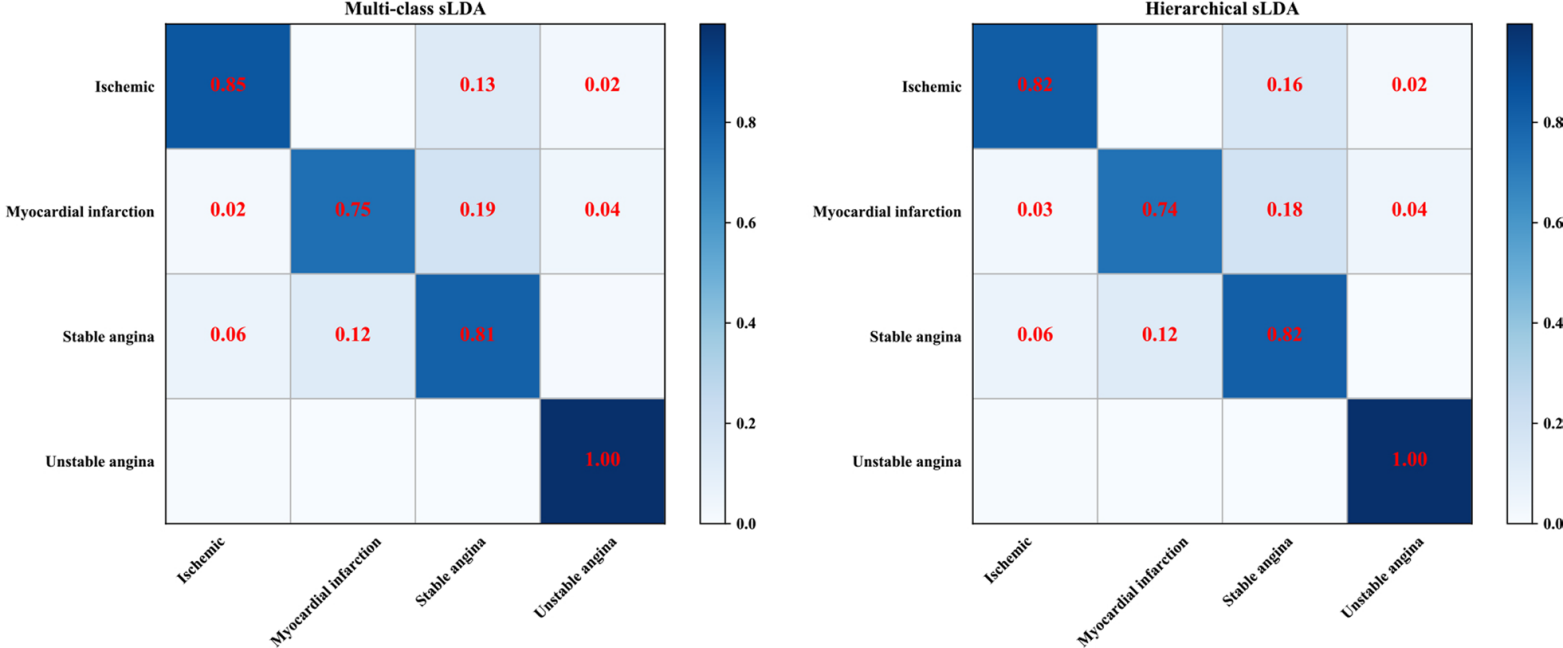
Comparison of confusion matrices. Comparison of confusion matrices of topic $K=70$; multi-class sLDA (left); Hierarchical sLDA (right)

The experiments were performed from topic $K = 10$ to $K = 70$ with intervals of 10. The results of accuracy, training and testing time are illustrated in Fig. 4 and the confusion matrices are shown in Fig. 5. Hierarchical sLDA model we proposed is competitive in both time performance and accuracy, as validated by experimental results. From Fig. 4-left and middle, comparison of over all classes based on fivefold cross validation it can be seen that Hierarchical sLDA ($K = 70$) reaches the average training time (669.69s) and testing time (0.32s) performance, which is 59.74% and 25.58% higher than the average training time (1663.42s) and testing time (0.43s) of Multi-class sLDA ($K = 70$) but almost no loss of accuracy on our datasets (See Fig. 4-right). The average accuracy of the Hierarchical sLDA ($K = 70$) is 74.53%, while that of Multi-class sLDA ($K = 70$) is 74.06%.

From Fig. 4-right, the average classification accuracy of the Hierarchical sLDA, as the number of topics increases, is smoothly converging, as well as not suffering from the overfitting problem. That means it provides more robust than other classifiers.

We evaluated on topic $K=70$ about different types of CHD using macro-F1 score, macro-Precision score, and macro-Recall score on test data respectively. A comparison between Hierarchical sLDA model and Multi-class sLDA model indicated that the two models are not significantly different(See Table 3).

**Table 3:** Comparison of macro-F1, Precision, Recall

	Macro-F1 (%)	Macro-Precision (%)	Macro-Recall (%)
Hierarchical sLDA	70.91	70.96	71.70
Multi-class sLDA	70.54	70.84	71.70

#### Risk stratification

Table 4 shows the top 5 risk factors of $K=70$ topics inferred by Hierarchical sLDA under high-, and low-risk tier separately of different types of CHD. The Hierarchical sLDA model of HDRs for CHD shows us:

- Different types of CHD generally have different risk factors, while Diabetes-Yes and Antiplatelet Medication-Yes are risk factors of Stable angina pectoris and Unstable angina pectoris separately. We can take a conclusion that Antiplatelet Medication provides an effective treatment, simultaneously should be highly aware of Diabetes.
- Uric acid-Abn. and SBP-Abn. are the risk factors of 4 types of CHD, which indicate the cause-and-effect correlation between these two risk factors and CHD.
- Both antiplatelet medication, ACEI/ARB and Lipid drug therapy are often used to reduce high-risk factors.
- Gender seems to have higher probability of different types of CHD.

**Table 4:** Risk factors of CHD extracted from Hierarchical sLDA

ICM	AMI	SAP	UAP
*High-risk*			
ST segment-Abn.	Hb-Abn.	SBP-Abn.	Diabetes-Yes
CTA stenosis-Mild	Duration-10 min	HbA1c-Abn.	Antiplatelet Medication-Yes
Carotid Atherosclerosis-Multiple plaque group	ST segment-Elevation	Uric acid-Abn.	HbA1c-Abn.
Fasting blood glucose-Abn.	$\beta$ blocker medications-Yes	ST segment-Change	Duration-3~5 min
Heart rate-Sinus velocity	Gender-Female	Lipid drug medication-Yes	Gender-Male
*Low-risk*			
CTA lesions-Single	CTA lesions-Single	Cardiac B-Ultrasound -Abn.	Hypertension-Yes
HbA1c-Abn.	ACEI/ARB medication-Yes	Hb-Abn.	DBP-Abn.
Age-45–65 years	Age-45–65 years	CTA stenosis-Mild	WBC-Abn.
Uric acid-Abn.	Uric acid-Abn.	Diabetes-Yes	SBP- Abn.
SBP- Abn.	SBP-Abn.	Antiplatelet medication-Yes	Uric acid-Abn.

#### Dirichlet hyperparameter

**Fig. 6:**
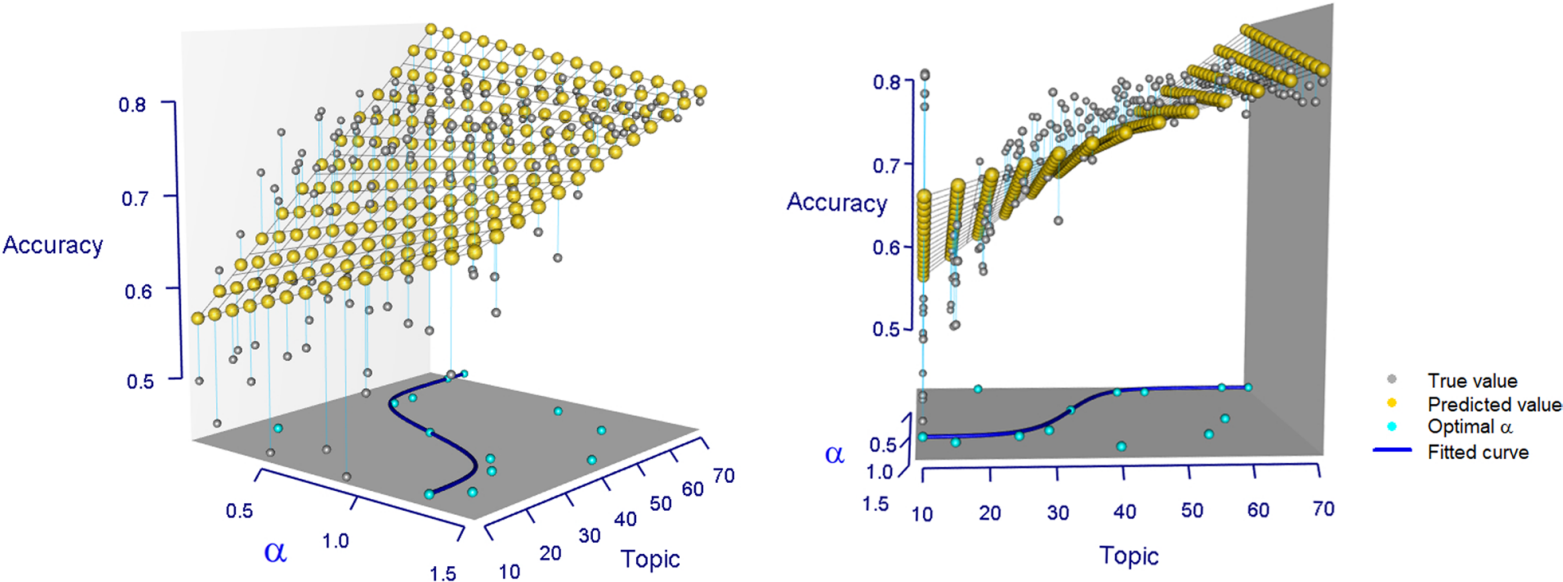
Selection of optimal hyperparameter $\alpha$. Using $\alpha$ from 0.1 to 1.5 with interval of 0.1, topic K from 10 to 70 with interval of 5, the 3-D representions show that we should optimize hyperparameter $\alpha$ with the fitted curve. The left view of 3-D represention (left); the front view of 3-D represention (right)

According to experience, Wei and Croft [32] pointed out that the choice of Dirichlet hyperparameter is not sensitive to the experimental results. They used symmetric Dirichlet priors in the estimation $\alpha ={50}/{K}$ and $\beta =0.01$. In this paper, according to our analysis (See “Parameter estimation” section) and the experimental results in Fig. 6, under the same topic (e.g., $K=70$), selecting different hyperparameter $\alpha$ has no significant effect on the average prediction accuracy (Fig. 6-right). However, after using $\alpha$ from 0.1 to 1.5 with interval of 0.1, K from 10 to 70 with interval of 5, the 3-D represention from Fig. 6-left and -right show that, rather than $\alpha ={50}/{K}$, we should optimize hyperparameter $\alpha$ with the fitted curve

$$\begin{aligned} \alpha = c_{1}+\frac{1}{1+exp{\left( c_{2}*\left( K-\frac{|V|}{2}\right) \right) }} \end{aligned}$$

where $c_{1}$ and $c_{2}$ are constants and |*V*| is the number of patient feature-value pairs (e.g., $c_{1} = 0.3$, $c_{2}$ = 0.25 and $|V| = 79$ in our experiments).

Panichella [33] also showed that search-based approaches are very effective in solving LDA hyperparameter tuning problem. In our proposed Hierarchical sLDA model, Dirichlet hyperparameter can affect the speed of convergence (See “Parameter estimation” section, Eqs. 10 and 11), therefore this structure has the potential to become a generic scheme for variants of sLDA-based model.

### Discussion

Traditional statistical models for risk stratification of CVD risk are well-developed, but typically less flexible than machine learning techniques and only hold under well controlled conditions for prediction and classification.

Hierarchical sLDA model is a variant of sLDA. The experimental results clearly demonstrate that the proposed Hierarchical sLDA has significant advantages on supervised CHD classification and risk stratification relative to the compared Multi-class sLDA approach, including accuracy and time performance.

Most classification techniques do not handle hierarchical features, which offer little insight beyond a flat feature-based segmentation, as they assume that features in the training datasets are fully independent. By categorizing patient features in HDRs as patient feature-value pairs, three-layer sLDA is improved to four-layer Hierarchical sLDA, which can accelerate the convergence of time-consuming Gibbs sampling.

We have shown that the model, as the number of topics increases, is converging smoothly. That means it is not suffering from overfitting problem and provides more robust than other classifiers.

Intriguingly, according to experience, Dirichlet hyperparameters are set to $\alpha = 50/$K and $\beta = 0.01$. However, we recommend that Dirichlet hyperparameter $\alpha$ may be optimized in another setup policy (See “Dirichlet hyperparameter” section). Through experiment analysis, there exists two limitations and weaknesses for the current approach:

- Insufficient training data. From the data source process, due to specific difficulties, features extraction is semi-automatic, which can be time and labor intensive. And therefore, Insufficient training data limits the performance of the model.
- More experiments. For future work, we will investigate the performance of our model when applied to other topic models and datasets.

## Conclusions

We hereby have proposed an approach in HDRs for risk stratification and classification of CHD simultaneously over our datasets, which is competitive in time performance and accuracy. Hierarchical processing of patient features can significantly improve the disadvantages of low efficiency and time-consuming Gibbs sampling of sLDA model. Meanwhile our model has the potential to be applied to other datasets by transforming the features of datasets into feature-value pairs. On the other hand, while coronary angiography is the gold standard for the diagnosis of CHD, but its limitations, such as invasive, random errors by the selection of radiographic projection, limit its wide clinical applications. Risk factors, which is extracted from risk stratification, can be used as a reference to provide individualized prevention and therapeutic decisions with non-invasive methods. However, the difficulty in processing complicated clinical applications suggests that this is still an open question needed to be solved in future research.

## Data Availability

The datasets used in this study and the source code of Hierarchical sLDA are available at https://github.com/flyingbiao/Hierachical-sLDA. All experiments were performed on Intel(R) Core(TM) i7 @4.20GHz with 16G bytes main memory running on Ubuntu 18.04.2. The algorithm was implemented using C++.
